# Osteo-Inductive Strategies for Enhancing Osseointegration and Optimizing Peri-Implant Emergence Profile: A Narrative Review

**DOI:** 10.3390/dj14050310

**Published:** 2026-05-18

**Authors:** Ioan Sirbu, Elisei Adelin Radu, Andy Radu Leibovici, Andreea Mihaela Custura, Ruxandra Stanescu, Alexandra Tuta, Vladimir Nastasie, Valentin Daniel Sirbu

**Affiliations:** 1 Discipline of Implanto-Prosthesis Therapy, Faculty of Dentistry, “Carol Davila” University of Medicine and Pharmacy, 37 Dionisie Lupu Street, District 2, 020021 Bucharest, Romaniaalexandra.tuta@umfcd.ro (A.T.);; 2 Discipline of Dental Prostheses Technology, Faculty of Dentistry, “Carol Davila” University of Medicine and Pharmacy, 37 Dionisie Lupu Street, District 2, 020021 Bucharest, Romania; 3 Doctoral School, Ovidius University of Constanța, 900470 Constanța, Romania; 4 Doctoral School, Faculty of Dentistry, “Carol Davila” University of Medicine and Pharmacy, 050474 Bucharest, Romania; vladimir.nastasie@drd.umfcd.ro

**Keywords:** osteoinduction, bone regeneration, osseointegration, dental implants, peri-implant soft tissue, guided bone regeneration, bioactive titanium surfaces

## Abstract

**Background:** Osteoinduction and bone regeneration are fundamental biological mechanisms enabling osseointegration and long-term durability of endosseous dental implants. In clinical practice, poor bone conditions, aesthetic demands, and peri-implant soft tissue problems commonly need the utilization of regenerative techniques targeted at optimizing both hard and soft tissue results. The purpose of this narrative review was to examine osteo-inductive and regenerative strategies currently employed in implant dentistry, with particular emphasis on the mechanobiological integration of hard–soft tissue regeneration and its implications for peri-implant tissue stability, osseointegration, and clinical predictability. **Methods:** A narrative literature review was done using PubMed and Scopus databases. Based on predetermined inclusion and exclusion criteria, studies published in English during the previous five years were reviewed. The core narrative analysis comprised a selection of physiologically relevant research that addressed osteo-inductive techniques, bone regeneration, osseointegration, and peri-implant soft tissue outcomes, as well as clinical studies, randomized controlled trials, systematic reviews, and narrative reviews. A narrative synthesis was carried out because of methodological variability. Special emphasis was placed on evidence addressing the biological and clinical interaction between hard- and soft-tissue regenerative strategies, reflecting the specific conceptual focus of the review. **Results:** The evidence presented suggests that implant surface biofunctionalization, biologically active grafting materials, guided bone regeneration, and supplementary biological treatments may have a favorable impact on implant stability and peri-implant bone healing. Several investigations also underlined the biological dependency between peri-implant bone regeneration and soft tissue architecture, stressing the significance of soft tissue thickness, keratinized mucosa, and emergence profile stability. Even in inflammatory environments, bioactive titanium surface changes showed osteogenic potential, indicating a supporting function in early osseointegration. **Conclusions:** By promoting osseointegration and improving peri-implant tissue outcomes, osteo-inductive and regenerative techniques are essential to modern implant dentistry; however, their greatest potential may lie in integrated hard–soft tissue regenerative approaches aimed at improving long-term clinical predictability. To further understand the clinical efficacy of combination hard–soft tissue regeneration methods, future well-designed clinical trials with standardized outcome measures are needed. Future research should further clarify the mechanobiological principles underlying these integrated regenerative approaches.

## 1. Introduction

One of the most dependable and widely acknowledged treatment modalities for the rehabilitation of partial and complete edentulism is dental implant therapy. The predictability and long-term success of implant-based rehabilitation have been substantially enhanced over the past few decades by ongoing advancements in biomaterials, surgical methods, and implant surface engineering. The initiation of sustained osseointegration, a physiologically complex process impacted by host-related variables, surgical procedures, implant design, and peri-implant tissue conditions, is crucial to the long-term success of endosseous dental implants [1,2]. Although modern implant systems have demonstrated high survival rates, achieving predictable clinical outcomes remains difficult in sites characterized by reduced bone volume, poor bone quality, or heightened aesthetic demands, particularly in the anterior area where tissue deficits may considerably affect both functional and aesthetic outcomes [2,3,4,5]. In order to improve peri-implant tissue conditions and increase the predictability of implant therapy, these clinical limitations have led to the development of biologically driven and regenerative methods.

In this regard, osteo-inductive and bone regenerative techniques to improve peri-implant healing and stability have received more attention. The ability of biologically functionalized implant surfaces, bioactive grafting materials, guided bone regenerative techniques (GBR), and the addition of angiogenic or osteogenic agents to enhance implant integration and stimulate bone formation has been studied [1,6,7,8]. This potential to actively modulate the bone–implant interface and induce early osteogenic responses has been further enhanced by developments in surface engineering, such as biomimetic mineralization and nanotopographical changes [6,7,8]. These strategies aim not only to promote early bone healing but also to create a more stable and biologically favorable implant–bone interface. However, the existing data is still inconsistent, and the therapeutic applicability of various methods differs depending on the indication, choice of material, and course of therapy [9,10,11]. Consequently, additional synthesis of the available evidence is necessary to clarify the clinical significance and prospective benefits of various osteo-inductive methodologies employed in implant dentistry.

In addition to hard tissue integration, peri-implant soft tissue conditions have become widely acknowledged as a crucial factor in implant success. Maintaining peri-implant health, reducing marginal bone loss, and achieving acceptable aesthetic results all depend on having a solid peri-implant mucosal seal, keratinized mucosa, and enough soft tissue thickness [12,13,14]. Recent clinical approaches have also demonstrated that modifications at the level of the supporting bone, including the use of guided bone regenerative abutments, can significantly influence gingival contour and emergence profile optimization in implant-supported restorations [12,13,14,15]. These findings further support the concept that hard tissue manipulation plays a critical role in shaping peri-implant soft tissue architecture and aesthetic outcomes [15]. The strong biological dependency between the hard and soft tissues surrounding dental implants has been highlighted by clinical investigations that show both bone regenerative treatments and soft tissue augmentation operations can affect peri-implant tissue dimensions and long-term stability [12,13,14,15,16,17,18,19]. As a result of this interaction, treatment concepts that simultaneously target peri-implant bone regeneration and soft tissue architecture are increasingly being explored. Despite these advances, limited synthesis exists regarding how hard- and soft-tissue regenerative strategies interact mechanobiologically and clinically to influence long-term implant stability, emergence profile optimization, and treatment predictability. This unresolved interface represents an important gap in contemporary implant literature.

Even though the research on peri-implant soft tissue preservation and bone regenerative techniques is expanding, these topics are often studied independently. This review specifically addresses this gap by prioritizing evidence at the interface between biological integration and clinical predictability. Variability in results and methodological discrepancies have been brought to light by systematic reviews and meta-analyses, especially when comparing various augmentation procedures or grafting materials [16,20]. The conversion of existing findings into standardized clinical procedures is thus limited by the incomplete synthesis of the combined impact of osteo-inductive therapies on osseointegration and the optimization of the peri-implant emergence profile [21,22]. Therefore, a thorough analysis of such approaches might advance knowledge of how regenerative techniques may influence both biological integration and aesthetic outcomes in implant therapy.

**Figure 1:**
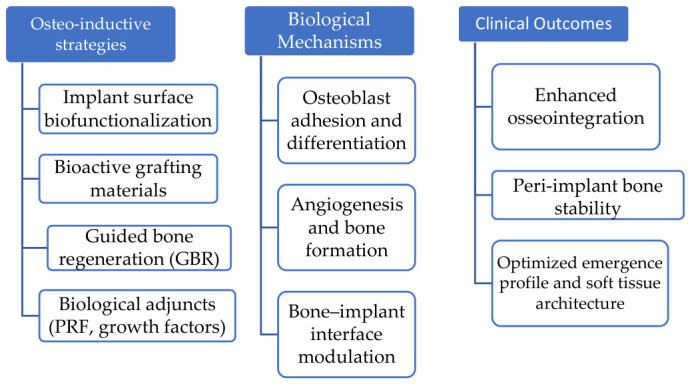
Conceptual synthesis model illustrating the interaction among osteo-inductive strategies, biological mechanisms, hard–soft tissue regeneration, and clinical predictability in implant dentistry.

With an emphasis on their effects on osseointegration and peri-implant soft tissue architecture, the objective of this narrative review is to critically examine osteo-inductive and regenerative strategies through a conceptual framework centered on the mechanobiological integration of hard–soft tissue regeneration and its implications for clinical predictability in implant therapy. This study aims to offer a thorough overview of current methods, highlight current limits, and propose future possibilities for increasing implant durability and aesthetic success by combining biological principles with clinical results. The conceptual relationships among osteo-inductive strategies, biological mechanisms involved in osseointegration, and their integration with peri-implant soft tissue stability as determinants of clinical predictability are summarized in Figure 1.

## 2. Materials and Methods

### 2.1. Search Strategy

The objective of this narrative literature review was to identify pertinent studies that addressed osteo-inductive and bone regenerative strategies in implant dentistry. The review was particularly focused on the impact of these strategies on peri-implant soft tissue architecture and osseointegration. The electronic databases PubMed and Scopus were searched in a structured manner to identify literature relevant to the conceptual scope of this narrative review.

The search was performed in PubMed using combinations of keywords related to dental implants, osseointegration, osteoinduction, bone regeneration, peri-implant soft tissues, and emergence profile (input: osteoinduction OR osteo-inductive OR “bone regeneration” OR “guided bone regeneration” OR PRF OR BMP) AND (“dental implant” OR “endosseous implant” OR osseointegration) AND (“peri-implant soft tissue” OR gingival OR “emergence profile”). A complementary search was subsequently conducted in Scopus using a simplified search strategy (input: osteo-inductive OR “bone regeneration” OR GBR) AND TITLE-ABS-KEY (“dental implant” OR osseointegration) AND TITLE-ABS-KEY (gingival OR “peri-implant soft tissue” OR “emergence profile”) adapted to database-specific constraints regarding the number of Boolean operators. The database search strings are reported to enhance reproducibility appropriate to a narrative review design. Both searches were restricted to articles published in the last five years and written in English. This time restriction was chosen because biologically driven implant therapies, bioactive materials, and regenerative protocols have evolved rapidly in recent years, and the review aimed to emphasize contemporary evidence with the greatest translational relevance. The database search was performed in January 2026. The search strategy was intended to support transparent literature identification within a narrative synthesis framework rather than to constitute a formal systematic review protocol.

The study selection process is illustrated in a flowchart presented in the Results Section.

### 2.2. Eligibility Considerations

Literature selection was guided by predefined eligibility considerations used to maintain thematic relevance and consistency with the conceptual focus of the review.

The following were the requirements for inclusion:

- Research on endosseous dental implants or regenerative techniques associated with implants;
- Studies assessing osteo-inductive or bone regenerative techniques, such as bioactive grafting materials, surface changes, guided bone regeneration, or biologically active adjuncts;
- Research presenting findings of implant stability, peri-implant bone behavior, or osseointegration;
- Clinical research, prospective or retrospective investigations, randomized controlled trials, and pertinent review papers offering clinical or biological insights;
- Articles published within the specified time period in peer-reviewed journals.

Studies focusing exclusively on in vitro models with limited clinical or translational relevance were excluded. Purely in vitro studies were not systematically included because the primary focus of this review was clinical and translational applicability; however, mechanistically relevant preclinical evidence was selectively incorporated when necessary to contextualize biological principles discussed in the review.

- Case reports or case series lacking generalizable clinical data;
- Articles about orthodontic mini-implants and other non-prosthetic implants;
- Research that mostly concentrated on oral surgery unrelated to implant treatment, peri-implant disease, or prosthetic results without regenerative implications;
- Publications published outside of the chosen publication period or not in English.

### 2.3. Data Extraction and Study Selection

Titles and abstracts of all records identified through the database searches were screened. Potentially suitable studies were next subjected to full-text review. Instead of performing a quantitative synthesis, the selection method sought to find publications pertinent to the review’s topic.

Data on publication year, research design, type of regenerative or osteo-inductive method, osseointegration assessment, evaluation of peri-implant soft tissue outcomes, and overall clinical relevance were recorded descriptively for each eligible study. The characteristics of each study and eligibility evaluation were methodically documented in an organized spreadsheet. Additional information regarding study objectives, regenerative techniques applied, clinical outcomes related to osseointegration, and peri-implant soft tissue parameters was also recorded when available.

To improve transparency and reduce selection subjectivity, literature screening and relevance assessment were independently conducted by two authors, with any differences resolved by discussion and consensus.

To enhance transparency, studies forming the core narrative synthesis were distinguished from complementary studies incorporated selectively to contextualize biological mechanisms or support interpretive discussion. These studies were not used to expand eligibility post hoc, but rather to support interpretive synthesis within the narrative framework. All cited studies were consistently listed in the References Section.

### 2.4. Methodological Strategy

A narrative synthesis technique was used due to the variation in study designs, materials, and outcome measures. No meta-analysis was performed. The chosen studies underwent core narrative analysis and were categorized based on their main areas of interest, which included combined hard–soft tissue methods, peri-implant soft tissue modulation, osteo-inductive tactics, and bone regenerative techniques. Due to the heterogeneity in study design, biomaterials, surgical protocols, and reported clinical outcomes, a quantitative meta-analysis was not considered appropriate. The present study was designed as a structured narrative review aligned with SANRA principles and intended to provide transparent interpretive synthesis rather than a formal systematic review or meta-analysis. Accordingly, PRISMA reporting standards and formal risk-of-bias assessment were not applied.

## 3. Results

### 3.1. Study Selection

**Figure 2:**
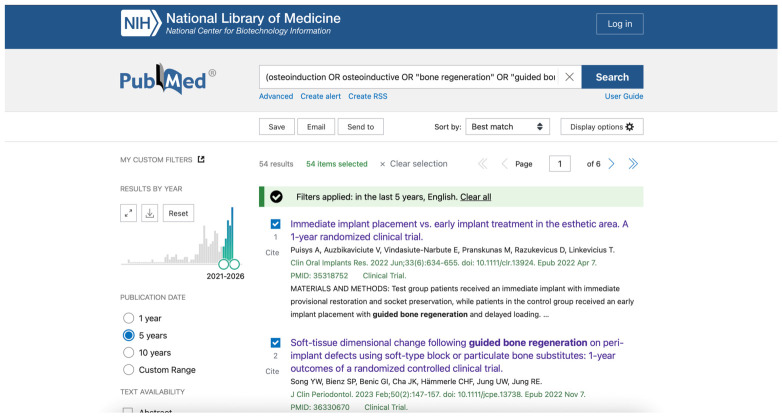
Representative documentation of the PubMed search strategy used to support literature identification.

**Figure 3:**
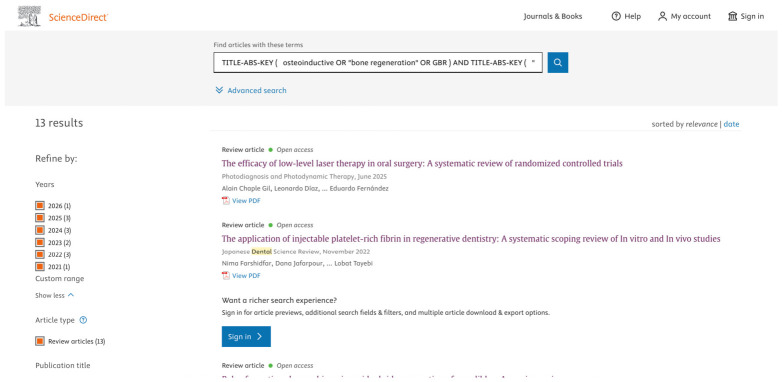
Representative documentation of the Scopus search strategy used to support literature identification.

A total of 67 documents were found by the electronic database search using PubMed and Scopus. While the Scopus search was used as a supplementary source to guarantee coverage of potentially pertinent studies not indexed in PubMed, the PubMed search produced most of the literature relevant to osteo-inductive and bone regenerating techniques in implant dentistry. Figure 2 and Figure 3 show a summary of the database search results.

**Figure 4:**
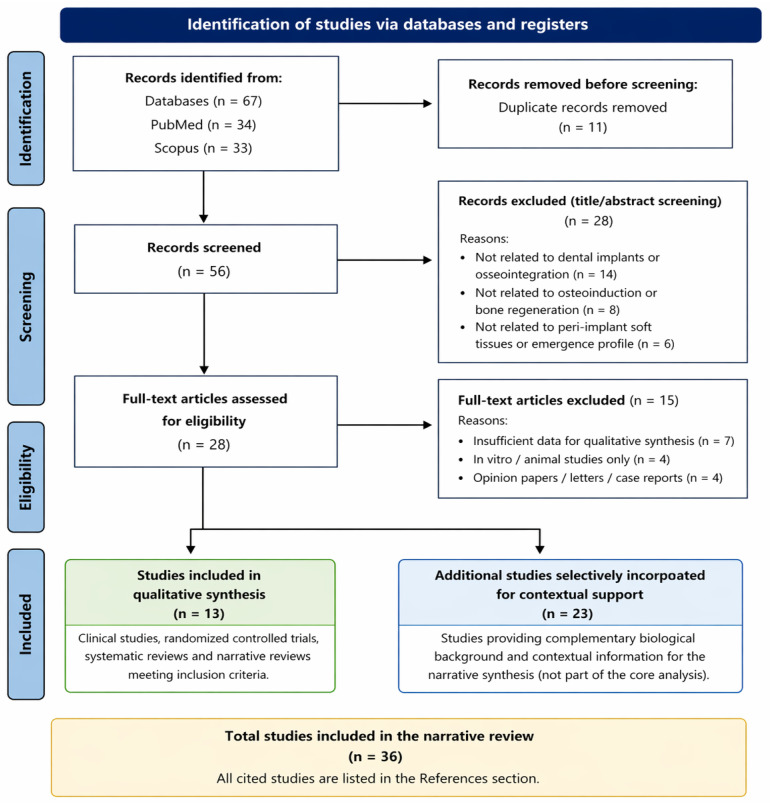
Flowchart illustrating the study selection process used in this narrative review, including database identification, title and abstract screening, full-text eligibility assessment, and inclusion of studies in the core narrative synthesis, together with complementary studies selectively incorporated for contextual interpretive support.

Figure 4 illustrates the study selection process and search strategy, including identification, screening, eligibility assessment, and inclusion of studies in the narrative synthesis.

Studies were screened based on title and abstract, followed by full-text eligibility assessment according to the predefined selection considerations. Articles unrelated to osteo-inductive or regenerative strategies in implant dentistry, peri-implant tissue outcomes, or osseointegration were excluded. Purely in vitro studies, isolated case reports without translational relevance, studies involving orthodontic mini-implants, and publications unrelated to implant-based regenerative therapy were also excluded.

Following full-text evaluation, 13 studies directly relevant to the central conceptual focus of the review were included in the core narrative synthesis. An additional 23 studies were selectively incorporated to provide complementary biological background and contextual interpretive support. The overall study selection process is illustrated in Figure 4.

A final group of papers was included for core narrative synthesis following full-text examination.

### 3.2. Features of the Included Research

Randomized controlled clinical trials, prospective and retrospective clinical studies, systematic and narrative reviews, and a few translational and preclinical studies directly related to implant dentistry were all included in the narrative synthesis. To improve peri-implant bone formation and stability, most of the included clinical investigations assessed guided bone regenerative techniques, bone grafting materials, biologically active adjuncts, or implant surface changes.

Results regarding peri-implant soft tissue behavior, such as dimensional alterations, soft tissue thickness, keratinized mucosa, and emergence profile stability, were also reported in several investigations. Although not solely implant-specific, a sample of publications was chosen to offer biological or mechanistic insight into osteo-inductive processes or regenerative materials.

Overall, the included literature reflected a growing interest in biologically driven regenerative approaches aimed at enhancing both peri-implant bone stability and soft tissue outcomes.

### 3.3. Descriptive Evidence Synthesis

The included studies were divided into the following thematic categories based on their focus: (i) enhancing osseointegration through osteo-inductive and bone regenerative strategies; (ii) modulating the bone–implant interface through surface-based and biomaterial-driven approaches; (iii) optimizing emergence profiles and peri-implant soft tissue augmentation; and (iv) combining hard- and soft-tissue regenerative protocols. These thematic categories reflect the main biological and clinical approaches currently explored to enhance osseointegration and optimize peri-implant tissue stability.

A quantitative meta-analysis was not possible because of the significant variability in research designs, materials, surgical procedures, and outcome variables. In order to connect biological principles with reported clinical outcomes and to find trends, consistencies, and gaps in the existing literature, a core narrative synthesis was carried out.

The principal osteo-inductive and regenerative strategies identified in the reviewed literature are summarized in Table 1.

## 4. Discussion

The findings of this narrative review highlight the increasing relevance of biologically driven regenerative strategies in contemporary implant dentistry and support a conceptual framework in which hard–soft tissue integration is viewed as a determinant of clinical predictability rather than as independent therapeutic domains. The reviewed literature suggests that multiple osteo-inductive approaches—including implant surface biofunctionalization, biologically active grafting materials, guided bone regenerative techniques, and biological adjuncts—may contribute to improved osseointegration and peri-implant tissue stability. The discussion is structured thematically to critically compare regenerative strategies, highlight methodological variability, and interpret their translational relevance in implant therapy. Where appropriate, greater interpretive emphasis was placed on higher-level evidence, including randomized clinical trials, prospective studies, and systematic reviews.

Section 4.1, Section 4.2, Section 4.3 and Section 4.4 also function as an integrative thematic synthesis model spanning biologic mechanisms, clinical strategies, and translational implications.

### 4.1. Techniques for Osteoinduction and Bone Regeneration That Support Osseointegration

Osteo-inductive and bone regenerative procedures constitute major drivers of peri-implant bone healing and long-term implant stability, particularly in clinical circumstances defined by poor bone conditions or increasing functional and aesthetic demands. According to the results of this narrative review, the most thoroughly studied strategies include the utilization of bioactive grafting materials, implant surface biofunctionalization, and guided bone regeneration (GBR) [15,23].

Implant surface engineering has become one of these tactics that shows the most promise for improving osseointegration. Biomimetic mineralization methods, nanotopographical surface alterations, and more recently, the immobilization of biofunctional molecules on titanium implant surfaces have been shown to influence protein adsorption, osteoblast adhesion, and osteogenic differentiation at the bone–implant interface, thereby enhancing early bone formation and increasing bone–implant contact [15,23,24].

Importantly, information covered in this review reveals that bioactive titanium surfaces, particularly those based on titanium dioxide (TiO_2_), display osteogenic potential even under inflammatory circumstances. Experimental studies indicate that TiO_2_-modified titanium surfaces can promote osteoblastic activity and osteogenic differentiation, suggesting a supporting function for osseointegration in physiologically challenging conditions. Recent advances in surface engineering have further demonstrated that multifunctional TiO_2_-based matrices, including fluoride-releasing and photothermal-responsive systems, can provide combined antibacterial effects, biosealing properties, and enhanced bone regeneration capacity [14,25]. These results have clinical significance since local inflammatory responses frequently accompany early implant recovery.

However, a number of influencing factors, such as host bone quality, surgical technique, and loading procedures, continue to affect clinical results. Therefore, rather than being a stand-alone factor that determines osseointegration, implant surface biofunctionalization should be considered an auxiliary technique [26]. In addition, recent randomized clinical evidence based on cone-beam computed tomography analysis has demonstrated that implant macro-design may significantly influence peri-implant bone remodeling in post-extraction sites, further emphasizing the multifactorial nature of osseointegration [27].

### 4.2. Biological Supplements and Regenerative Substances

Platelet concentrates and growth factor-based treatments are two examples of biological adjuncts that are being used more often in implant dentistry regenerative procedures. These biologic agents may stimulate bone regeneration and early healing surrounding dental implants by promoting angiogenesis, cellular proliferation, and early osteogenic stimulation, as demonstrated in recent clinical studies evaluating the use of platelet concentrates in combination with grafting materials during immediate implant placement [27,28,29]. In particular, injectable platelet-rich fibrin (I-PRF) has shown promising results in enhancing regenerative outcomes when used as an adjunct to bone graft materials [29]. However, the formation of standardized clinical recommendations is constrained by considerable variability in preparation protocols, application techniques, and reported outcomes. Nevertheless, recent clinical studies have demonstrated that the use of platelet-rich fibrin may positively influence both primary and secondary implant stability, suggesting a beneficial role in early implant integration [30,31,32]. Furthermore, clinical evidence indicates that the use of platelet-rich fibrin and concentrated growth factors in immediate implant placement, even in the presence of periapical lesions, can enhance healing outcomes and support favorable peri-implant tissue conditions over time [33].

Similarly, regenerative materials and biologically active strategies that can enable bone augmentation before or in combination with implant insertion have been proposed, including dentin-derived grafts and other biologically active scaffolds. Preclinical evidence has demonstrated that osteo-inductive molecules such as bone morphogenetic proteins (e.g., BMP-9) can significantly enhance bone formation and support alveolar ridge preservation following tooth extraction [34,35]. Dentin-derived materials may have advantageous osteoconductive and maybe osteo-inductive qualities, supporting ridge preservation and augmentation results, according to systematic evaluations [28]. Nevertheless, their unique contribution to long-term osseointegration remains difficult to differentiate from the impact of surgical technique and host-related variables. Moreover, variability in preparation protocols and outcome reporting across studies may partly explain inconsistencies regarding the magnitude of their regenerative effects.

### 4.3. Managing Soft Tissue Around Implants and Improving Emergence Profiles

The reviewed research highlights the importance of soft tissue architecture in achieving stable biological and aesthetic implant results, going beyond peri-implant bone regeneration. Clinical investigations consistently reveal that appropriate soft tissue thickness, sufficient keratinized mucosa, and peri-implant mucosal stability are linked with lower marginal bone loss and improved long-term peri-implant health [2,9,13]. Both soft tissue augmentation procedures and regenerative bone approaches have been demonstrated to influence peri-implant tissue dimensions, highlighting the biological interdependence between hard and soft tissues. Recent evidence suggests that soft tissue grafting procedures, such as free gingival grafts, may also exert osteo-inductive effects on the underlying alveolar bone, further supporting this functional relationship [13,36,37]. In addition, combined approaches using free gingival grafts and xenogeneic collagen matrices following vertical bone augmentation have been shown to effectively increase keratinized tissue and improve peri-implant soft tissue conditions [38]. Furthermore, randomized clinical evidence has demonstrated that both free gingival grafts and platelet-rich fibrin membranes can significantly enhance the width of keratinized mucosa around dental implants, although differences in biological behavior and clinical handling may influence treatment selection [39].

Importantly, peri-implant bone repair and soft tissue maintenance should not be treated as independent treatment aims. While inadequate soft tissue conditions may worsen peri-implant bone remodeling and biological problems, inadequate underlying bone support may jeopardize soft tissue stability and emergence profile maintenance [2,13,32]. These findings highlight the need for comprehensive treatment planning, especially in areas with high aesthetic standards. Advanced clinical approaches integrating hard and soft tissue management, such as the combination of guided bone regeneration with root submergence techniques, have been proposed to preserve tissue architecture and optimize aesthetic outcomes in challenging cases [40].

### 4.4. Methods of Combined Hard–Soft Tissue Regeneration

The implementation of integrated hard–soft tissue regenerative procedures is an emerging idea in current implant dentistry. In challenging clinical situations, including immediate implant placement, buccal bone dehiscence defects, and thin tissue phenotypes, concurrent bone regeneration and soft tissue management may provide synergistic advantages, as supported by clinical and systematic evidence. In this context, techniques such as dual-zone bone grafting in immediate implant and immediate restoration protocols have been shown to support peri-implant tissue stability and optimize gingival contour, contributing to improved aesthetic outcomes [41].

The use of collagen-based matrices in the treatment of peri-implant dehiscence defects has also demonstrated favorable clinical outcomes, contributing to both hard and soft tissue regeneration [42]. Recent studies have also demonstrated that surgical approaches, such as flap design and membrane coverage techniques, can significantly influence healing outcomes following guided tissue regenerative procedures [32,43,44]. In particular, differences between open and closed flap techniques may impact membrane stability, soft tissue healing, and ultimately peri-implant tissue outcomes [44]. Heterogeneity in research design, sample size, and outcome reporting, however, continues to constrain the available data [15,20]. In addition, recent clinical evidence suggests that complication rates, particularly membrane exposure and dehiscence in guided bone regenerative procedures using titanium meshes, may be influenced by patient-related and site-specific factors such as smoking status and defect location, further complicating outcome predictability [45]. Nevertheless, clinical studies using advanced barrier membranes and occlusive systems, such as dehydrated amnion–chorion membranes and titanium occlusive barriers, as well as biologically derived membranes, have demonstrated favorable regenerative outcomes and stable peri-implant conditions in both vertical and horizontal bone augmentation procedures. Recent randomized clinical evidence further indicates that the use of biologically derived membranes, such as small intestine submucosa, may enhance bone regeneration and support early loading protocols in implant therapy, particularly in the anterior maxillary region [15,20,46,47,48].

The absence of standardized regenerative procedures and unified outcome measures limits direct comparison between research and restricts the development of evidence-based treatment guidelines. While lower-level evidence often supports biological reasoning, randomized clinical evidence provides stronger support for the clinical predictability of these approaches. Nevertheless, high-level randomized clinical evidence has demonstrated that the use of guided bone regeneration in the management of buccal dehiscence defects may lead to improved peri-implant outcomes compared to non-regenerative approaches [49]. These findings highlight the clinical relevance of GBR techniques, particularly in defect-related scenarios where spontaneous healing may be insufficient. Future research should prioritize well-designed randomized clinical trials investigating integrated regenerative methods employing defined biological, radiological, and clinical objectives.

### 4.5. Clinical Implications and Limitations of the Existing Evidence

Despite the increasing number of publications addressing regenerative strategies in implant dentistry, several knowledge gaps remain. In particular, the heterogeneity in study design, biomaterials used, and outcome assessment methods limits the possibility of establishing standardized clinical protocols.

From a clinical point of view, the existing data supports the use of osteo-inductive and regenerative methods as part of a patient-specific and defect-oriented approach to implant treatment. Clinical implications discussed in this review were interpreted with particular emphasis on higher-level evidence, including randomized clinical trials, prospective studies, and systematic reviews. When choosing regenerative therapies, clinicians should carefully examine soft tissue phenotype, bone quality, defect shape, and aesthetic considerations. Crucially, no one osteo-inductive method can provide better soft tissue results or improved osseointegration in every situation [1,37], underscoring the need for surgical skill and thorough treatment planning. Future research should also explore the interaction between regenerative bone procedures and soft tissue phenotype in order to better understand their combined influence on peri-implant aesthetics and emergence profile stability. Long-term clinical evidence further supports the safety and effectiveness of advanced regenerative approaches, including the use of autologous stromal vascular fraction in combination with calcium phosphate biomaterials, which have demonstrated stable outcomes over extended follow-up periods of up to 10 years [50]. Such findings emphasize the importance of evaluating not only short-term regenerative success but also long-term stability and biological safety when selecting treatment strategies.

The narrative design of this review imposes numerous constraints with respect to its scope. Quantitative synthesis and meta-analysis were not done due to heterogeneity in study designs, materials, and outcome measures. Additionally, although the careful inclusion of physiologically relevant research helps contextual knowledge, it may restrict direct comparability. Nevertheless, the straightforward eligibility evaluation and organized synthesis increase the interpretive value of the present review.

## 5. Conclusions

The results of this narrative review demonstrate that osteo-inductive and bone regenerative techniques are essential for promoting osseointegration and guaranteeing the long-term stability of endosseous dental implants. When properly chosen and used as part of an all-encompassing treatment strategy, techniques including guided bone regeneration, the use of biologically active grafting materials, implant surface biofunctionalization, and supplementary biological treatments all contribute to improved peri-implant bone healing.

Because soft tissue stability and peri-implant bone regeneration are physiologically linked and jointly affect functional and aesthetic results, the evidence reviewed in this article emphasizes that integrated hard–soft tissue regeneration should be viewed as a determinant of clinical predictability rather than as separate therapeutic objectives. In particular, treatments that promote osteogenic activity at the bone–implant interface, including bioactive titanium surface changes, may give additional advantages in challenging clinical situations, such as locations defined by inflammatory conditions or low bone supply.

It is not possible to advocate a single osteo-inductive method despite promising clinical and biological results. Rather, the evidence supports individualized regenerative strategies guided by defect-specific and biologically integrated treatment planning. Treatment effectiveness largely depends on patient-specific variables, defect morphology, surgical competence, and prosthetic design. The variability in the study design and outcome measures within the available literature prevents direct comparison and the establishment of uniform therapeutic approaches.

Long-term prospective studies and well-designed randomized clinical trials assessing integrated hard–soft tissue regenerative techniques with defined biological, radiological, and clinical outcomes should be the focus of future research. Such studies are necessary to better understand the mechanobiological principles underlying integrated regenerative approaches and their role in maximizing osseointegration and achieving predictable, long-term implant success.

## Figures and Tables

**Table 1:** Comparative synthesis of the principal osteo-inductive and regenerative strategies discussed in this review and their biological mechanisms and clinical applications in implant dentistry.

Strategy	Biological Mechanism	Clinical Purpose	Level of Evidence	Main Limitations/Considerations
Implant surface biofunctionalization (nanotopography, biomimetic coatings)	Enhances protein adsorption, osteoblast adhesion, and osteogenic differentiation	Accelerates early osseointegration and increases bone–implant contact	Experimental studies, clinical studies, systematic reviews	Outcomes influenced by host factors and surgical variables
Guided bone regeneration (GBR)	Barrier membranes allow selective bone growth while excluding soft tissue cells	Reconstruction of peri-implant bone defects and ridge augmentation	RCTs, prospective studies, systematic reviews	Technique sensitivity, membrane complications, protocol heterogeneity
Bioactive grafting materials (xenografts, allografts, dentin-derived grafts)	Provide osteoconductive scaffold and may stimulate osteogenesis	Bone augmentation and ridge preservation	Clinical studies, RCTs, systematic reviews	Biomaterial-dependent variability
Platelet concentrates (PRF, CGF)	Release growth factors that stimulate angiogenesis and early bone healing	Adjunctive enhancement of bone regeneration around implants	Clinical studies, RCTs	Lack of protocol standardization
Bioactive titanium surfaces (e.g., TiO_2_ nanotubes)	Promote osteoblastic activity and bone formation at the implant interface	Improved osseointegration in challenging conditions	Experimental + translational evidence	Limited long-term comparative clinical data
Combined hard–soft tissue regenerative approaches	Integrate bone regeneration with soft tissue augmentation	Improved peri-implant tissue stability and emergence profile	Clinical studies, RCTs, long-term cohort evidence	Heterogeneous protocols and outcomes

## Data Availability

The original contributions presented in this study are included in the article. Further inquiries can be directed to the corresponding authors.
